# Abscisic Acid Negatively Modulates Heat Tolerance in Rolled Leaf Rice by Increasing Leaf Temperature and Regulating Energy Homeostasis

**DOI:** 10.1186/s12284-020-00379-3

**Published:** 2020-03-13

**Authors:** Guangyan Li, Caixia Zhang, Guangheng Zhang, Weimeng Fu, Baohua Feng, Tingting Chen, Shaobing Peng, Longxing Tao, Guanfu Fu

**Affiliations:** 1 National Key Laboratory of Rice Biology, China National Rice Research Institute, Hangzhou, 310006 Zhejiang China; 2 Crop Production and Physiology Center (CPPC), College of Plant Science and Technology, Huazhong Agricultural University, Wuhan, 430070 Hubei China; 3 State Key Laboratory of Crop Biology and College of Agronomy, Shandong Agricultural University, Tai’an, 271018 Shandong China

**Keywords:** *Oryza sativa* L., Rolled leaf, Heat stress, Abscisic acid, Tissue temperature, Energy homeostasis

## Abstract

**Background:**

Abscisic acid (ABA) acts as a signaling hormone in plants against abiotic stress, but its function in energy homeostasis under heat stress is unclear.

**Results:**

Two rice genotypes, Nipponbare (wild-type, WT) with flat leaves and its mutant *high temperature susceptibility* (*hts*) plant with semi-rolled leaves, were subjected to heat stress. We found significantly higher tissue temperature, respiration rate, and ABA and H_2_O_2_ contents in leaves as well as a lower transpiration rate and stomatal conductance in *hts* than WT plants. Additionally, increased expression of *HSP71.1* and *HSP24.1* as well as greater increases in carbohydrate content, ATP, NAD (H), and dry matter weight, were detected in WT than *hts* plants under heat stress. More importantly, exogenous ABA significantly decreased heat tolerance of *hts* plants, but clearly enhanced heat resistance of WT plants. The increases in carbohydrates, ATP, NAD (H), and heat shock proteins in WT plants were enhanced by ABA under heat stress, whereas these increases were reduced in *hts* plants.

**Conclusion:**

It was concluded that ABA is a negative regulator of heat tolerance in *hts* plants with semi-rolled leaves by modulating energy homeostasis.

## Background

Abscisic acid (ABA) is an important phytohormone controlling many developmental and physiological processes under natural and stressed conditions (Verslues and Zhu 2007; Huang et al. 2017). ABA accumulates in the developing embryo where it regulates seed development, seed maturation, and seed dormancy (Verslues and Zhu 2007). It also acts as a signaling molecule to defend against biotic stress including pathogen infection (Mittler and Blumwald 2015; Kang et al. 2017). Under drought stress, ABA accumulates to induce closing of leaf stomata to reduce water loss from plants (Guajardo et al. 2016), and enhances antioxidant capacity, heat shock proteins (HSPs), the sugar response, and accumulation processes for plants to tolerate desiccation (Guajardo et al. 2016; Yao et al. 2018; Jahan et al. 2019). Expression of the CBF/DREB1 transcription factors and cold-hardiness increase in grapevine dormant buds in response to ABA when the plants are subjected to cold stress (Rubio et al. 2018; Albertos et al. 2019). Additionally, ABA also functions in the heat stress response in plants, but the underlying mechanism remains unclear (Suzuki et al. 2016; Zandalinas et al. 2016; Zhang et al. 2017).

Heat stress is defined as a rise in temperature beyond a critical threshold or period of time that can cause irreversible damage (Wahid et al. 2007; Zandalinas et al. 2018; Sehgal et al. 2019). Thermolabile proteins can be inactivated, reactive oxygen species (ROS) accumulate, and programmed cell death is induced in plants during this process (Grover et al. 2013; Liu et al. 2016; Zhang et al. 2016). Such stressors occurring during the reproductive stage significantly decrease grain yield (Feng et al. 2018; Zhang et al. 2018). It has been reported that ABA enhances heat tolerance in plants by increasing H_2_O_2_ levels to induce antioxidant capacity and HSPs (Li et al. 2014; Islam et al. 2019). Additionally, ABA accumulates to enhance ascorbate peroxidase 1 and multiprotein bridging factor 1 activities to protect against heat damage in plants (Zandalinas et al. 2016). In contrast, a decrease in endogenous ABA level has been detected in plants sprayed with an ABA biosynthesis inhibitor, which impairs heat tolerance (Ding et al. 2010; Kumar et al. 2012). Importantly, heat-induced damage is more severe in ABA-deficient mutants than in their parental cultivars (Wang et al. 2014; Wu et al. 2017). It is worth noting that most of the current stress research is focused on understanding the ABA signaling pathway in heat tolerance of plants, and very few studies have been conducted on energy homeostasis in plants under heat stress.

Maintaining energy homeostasis is a challenge for all living organisms, and an intimate relationship exists between energy availability and stress tolerance in plants (De Block and Van Lijsebettens 2011; Dröge-Laser and Weiste 2018). Plant growth and development are inhibited or plants die if the extreme stress lasts until an energy threshold is reached at which time the damage caused by the stress can no longer be repaired (Baena-González and Sheen 2008). Unfortunately, the enhancing HSPs and antioxidant capacity is a high energy consumption process in plants under stress (Flaherty et al. 1990; Grenert et al. 1997; Zhu 2016). Therefore, the accumulation of HSPs and enhancement of antioxidant capacity induced by ABA under heat stress might be related with its ability to maintain energy homeostasis or enhance energy-use efficiency (De Block and Van Lijsebettens 2011). This hypothesis was confirmed by the results of Islam et al. (2019), who reported that ABA enhances sucrose transport and metabolism to prevent depletion of ATP and maintain energy homeostasis in rice spikelets, and thus heat resistance.

However, negative modulation of ABA has also been detected in plants responding to abiotic or biotic stress. ABA increases susceptibility to a plant virus by modulating the rice-RBSDV interaction, suppressing the jasmonate pathway, and regulating ROS levels (Xie et al. 2018). When plants are exposed to heat stress, 10 μmol·L^− 1^ ABA greatly enhances heat tolerance, while tolerance was reversed in the 100 μmol·L^− 1^ ABA treatment (Robertson et al. 1994; Gong et al. 1998; Li et al. 2014). Interestingly, rice plants sprayed with 100 μmol·L^− 1^ ABA attain higher pollen viability under heat stress at the pollen mother cell meiotic stage (Islam et al. 2019). These findings suggest that the negative effects of ABA shown in plants against stress are not only due to the high concentrations, but also the plant types, such as rolled leaf plants. Lower transpiration ability and higher leaf temperature are often found in rice plants with rolled leaves (unpublished data). This suggests that rolled leaf plants consume more energy due to a higher respiration rate under heat stress. In this case, more damage would be observed in rice plants with rolled leaves when sprayed with ABA under heat stress, possibly because ABA induces stomatal closure, resulting in lower net photosynthetic and transpiration rate but higher leaf temperatures (Islam et al. 2018). It has been inferred that more energy is required in rice plants sprayed with ABA. However, the relationship between ABA and energy homeostasis in rolled leaf plants has not been documented under heat stress.

In this study, more damage was found in mutant plants with semi-rolled leaves (*high temperature susceptibility; hts*) than its wild-type Nipponbare (WT) plants caused by heat stress. Higher leaf temperature and ABA content were observed in *hts* than WT plants under heat stress. Accordingly, greater increases in ATP and NAD (H) contents were found in WT than *hts* plants under heat stress compared with their respective controls. Thus, we wondered whether ABA was negatively involved in heat acclimation by regulating energy homeostasis in rolled leaf plants as well as the heat damage exacerbated by exogenous ABA.

## Materials and Methods

### Plant Materials and Growth Conditions

This study was conducted at the experimental farm of the China National Rice Research Institute, Hangzhou, Zhejiang Province, China. Two rice genotypes, WT and its mutant *hts*, were selected for use in this study. The *hts* semi-rolled leaf mutant was isolated from the ethyl methane sulfonate-induced japonica rice Nipponbare mutant bank (Zhang et al. 2018). This mutant has been self-pollinated for more than nine generations, and the semi-rolled leaf phenotype has been stably expressed in the greenhouse and field. The rice seeds were directly sown in pots (10 cm height and 10 cm diameter) in a plant growth chamber with an automatic temperature control system and controlled relative humidity until the five to six leaf stage.

### The Response of Rice Plants to Different Temperature Stressors

At the five to six leaf stage, WT and *hts* plants were subjected to different temperatures, such as 30 °C, 35 °C, and 40 °C for 72 h, while the rice plants were only subjected to 45 °C for 24 h. During the stress period, the temperature was constant, and the relative humidity was 70% under natural sunlight conditions. The first fully expanded leaves were collected at the end of the heat stress period to determine maximum fluorescence quanta of PSII (Fv/Fm) and leaf ion leakage, as well as malondialdehyde (MDA) and chlorophyll contents.

### The WT and *hts* Plant Responses to Heat Stress

According to the above results, the five to six leaf stage of WT and *hts* plants was subjected to heat stress at 40 °C for 72 h. The control temperature was 30 °C. The temperatures were constant during the stress period, and relative humidity was 70% under natural sunlight conditions. Additionally, the first fully expanded rice leaves were collected about 4 h, 10 h (at night), 28 h, 34 h (at night), 48 h, and 72 h after the heat stress to determine the contents of carbohydrates, ABA, H_2_O_2_, NAD (H), and ATP as well as the relative expression levels of genes related to sucrose transport and metabolism, poly-ADP ribose polymerase (PARP) and HSPs. Additionally, leaf temperature, stomatal conductance, as well as transpiration and respiration rates were determined.

### Effect of ABA on Rice Plants under High Temperature Conditions

According to the above results, ABA accelerated heat injury of rice plants with rolled leaves under heat stress. Therefore, different ABA concentrations, such as 1 μmol·L^− 1^, 10 μmol·L^− 1^, and 100 μmol·L^− 1^, were used in this experiment. Chemicals containing 0.1% (v/v) Tween20 as the surfactant were foliar applied at 9:00 a.m. at a volume of 10 mL per pot about 30 min before the heat stress. About 24 h later, leaf temperature during the day (28 h) and night (34 h), stomatal conductance, and transpiration rate were determined. The Fv/Fm value of the leaves was determined when the heat stress ended. The samples of the first fully expanded leaves were collected to determine the content of carbohydrates, NAD (H), and ATP as well as the relative expression levels of genes related to sucrose transport and metabolism, PARP and HSPs.

### Measurement of Chlorophyll Content, Chlorophyll Fluorescence Parameters, and Gas Exchange

Chlorophyll was extracted according to the method described by Sartory and Grobbelaar (1984). About 0.1 g of leaf sample was cut into pieces and immersed in 20 mL of 95% ethanol in the dark for 48 h. Chlorophyll concentration was determined at 665 nm and 649 nm using a spectrophotometer (Lambda25; Perkin Elmer, Freemont, CA, USA). Chlorophyll *a* and *b* values were calculated with the following equations: C_a_ (μg·mL^− 1^) = 13.95 × (A665) − 6.88 × (A649), C_b_ (μg·mL^− 1^) = 24.96 × (A649) − 7.32 × (A665).

Chlorophyll fluorescence was measured with a portable chlorophyll fluorescence spectrometer (PAM-2500 chlorophyll fluorescence system; Heinz Walz, Effeltrich, Germany). The Fv/Fm values were determined after a 30-min dark adaptation period.

Stomatal conductance and transpiration rates were analyzed using a Li-COR 6400 portable photosynthesis system (Li-COR Biosciences Inc., Lincoln, NE, USA) under the following conditions: photosynthetic photon flux density of 1200 μmol·m^− 2^·s^− 1^; ambient CO_2_ (400 μmol·mol^− 1^); 6 cm^2^ leaf area; 500 μmol·s^− 1^ flow speed, and temperature according to the treatment.

The respiration rate of the leaves was also analyzed at night (2 h after dark) using the Li-COR 6400 portable photosynthesis system under the following conditions: photosynthetic photon flux density of 0 μmol·m^− 2^·s^− 1^; ambient CO_2_ (400 μmol·mol^− 1^); 6 cm^2^ leaf area; 500 μmol·s^− 1^ flow speed, and temperature according to the treatment.

### Dry Matter Weight Measurement

The rice plants were sampled to determine dry matter weight and its distribution at the end of the heat stress. The plants were divided into leaf and sheath-stems, and were dried at 85 °C for 48 h and weighed.

### Carbohydrate Measurement

According to the methods of DuBois et al. (1956) with some modifications, about 0.5 g of frozen leaves was extracted with deionized water. The concentration of starch was determined at 620 nm using a spectrophotometer. The extracting method for sucrose, glucose, and fructose was similar to that of soluble sugars. Sucrose, glucose, fructose and soluble sugar contents were determined by the methods of Zhang (1977) with some modifications. Total non-structural carbohydrate (NSC) content was considered the sum of the soluble sugar and starch contents.

### Thermal Imaging of Rice Plants

According to the method of Zhang et al. (2016), the temperatures of the rice plants were determined from 9:30 to 10:30 during the day and at night (19:30 to 20:30) using an FLIR Therma CAM™ S65 system (FLIR Systems Inc., Portland, OR. USA) with a wide-angle camera lens (18 mm IR-LENS). The camera was set up 1.0 m away from the rice plants. A black cloth was set up behind the rice plants to minimize interference from other sources when recording the temperature. The data were analyzed with Therma CAM Researcher Pro 2.7 software (FLIR Systems).

### H_2_O_2_ Measurement

H_2_O_2_ content was determined according to the method of Brennan and Frenkel (1977) with some modifications. About 0.2 g of frozen leaves were homogenized in 4 mL of 10 mmol·L^− 1^ 3-amino-1,2,4-triazole (not cold acetone), and then centrifuged for 25 min at 6000×*g*. Thereafter, 1 mL 0.1% titanium tetrachloride dissolved in 20% H_2_SO_4_ was added to 2 mL of the supernatant. The reaction solution was further centrifuged to remove undissolved materials, and absorbance was recorded at 410 nm.

### Lipid Peroxidation Measurements

According to the method of Dhindsa et al. (1981), the concentration of thiobarbituric acid reactive substances was determined to estimate MDA content. About 0.2 g of frozen leaves were homogenized in 2 mL of 5% trichloroacetic acid for this process.

### Relative Electrical Conductance Measurement

Relative electrical conductance (REC) was measured by the method of Xiong et al. (2012). About 0.5 g fresh rice leaves were collected quickly at the end of the heat stress, cut into 25-mm^2^ pieces, and added to a test tube with 12 mL deionized water for 2 h at 25 °C. The electrical conductivity of the solution (EC1) was determined with a conductivity meter (DDA-11A; Shanghai Hongyi Instrument Co., Ltd., Shanghai, China) after the incubation. The samples were heated at 80 °C for 2 h in their effusates and cooled to 25 °C, and electrical conductivity (EC2) was measured again in the bathing solution. Ion leakage was calculated as the ratio between EC1 and EC2.

### Abscisic Acid Measurements

About 0.2 g of frozen leaves were weighed and an enzyme-linked immunosorbent assay was used to determine endogenous ABA content according to the method of Yang et al. (2001). All procedures were conducted in accordance with the manufacturer’s instructions (China Agricultural University, Beijing, China).

### Measurement of ATP and NAD (H) Contents

Frozen leaves (0.1 g) were homogenized with 2 mL of perchloric acid in an ice bath and centrifuged for 10 min at 8000×*g*. Then, 2 mL of NaOH was added to the supernatant and centrifuged for 10 min at 8000×*g*. The supernatant was collected and placed in an ice bath for analysis. An ATP assay kit was used to determine ATP content according to the manufacturer’s instructions (Comin Biotechnology Co., Ltd., Suzhou, China).

According to the method of Matsumura and Miyachi (1983), NAD^+^ and NADH were extracted with 1 mL 0.1 mol·L^− 1^ HCl and 0.1 mol·L^− 1^ NaOH, respectively. An assay kit was used to determine the NAD^+^ and NADH contents, according to the manufacturer’s instructions (Comin Biotechnology Co., Ltd.).

### Leaf Water Potential Measurements

Leaf water potential was determined according to the method of Chen et al. (2018). The youngest fully expanded leaves were collected 34 h after heat stress to determine the water potential using a PSYPRO Dew Point Microvoltmeter equipped with a leaf sample chamber (Model C-52, Wescor Co., Logan, UT, USA). Discs of leaves were obtained with a piercer, and placed inside the measurement chamber. The data were recorded after 30-min equilibrium of the microenvironment in the measurement chamber.

### Morphology of Bulliform Cells and Leaf Stomata

The leaf samples used for observing the morphology of bulliform cell were collected from the youngest fully expanded leaves under control conditions. The sections were prepared with a freezing microtome, and then the tissue slices of the bulliform cells were photographed with a fluorescence microscope (DM4000; Leica, Jena, Germany). The area of the bulliform cells was measured using Image J software (National Institutes of Health, Bethesda, MD, USA) by selecting the calculation tool for irregular delineation.

The number of stomata, their opening rate, and their aperture were determined according to the method of Feng et al. (2018), that the youngest fully expanded leaves of the rice plants were detached and immediately immersed in 3.5% glutaraldehyde in phosphate buffer (0.1 M, pH = 7.0) at 4 °C. These samples were dehydrated in a Hitachi Model HCP-2 critical point dryer, coated with gold-palladium in the Hitachi Model E-1010 ion sputter for 4–5 min, and observed with a Hitachi Model SU-8010 scanning electron microscope.

### Quantitative Real-Time Polymerase Chain Reaction (PCR) Analysis

Total RNA was extracted from 100 mg of leaves using the TRIpure reagent (Aidlab Biotechnologies, Beijing, China). RNA was converted to first-strand cDNA using the ReverTra Ace qPCR RT Master Mix (TOYOBO, Shanghai, China). The resulting cDNA was used as the template for quantitative PCR amplification in a Thermal Cycler Dice Real Time System II (TaKaRa Biotechnology, Dalian, China) using SYBR Green I (TOYOBO) as the fluorescent reporter. The primers were designed using PRIMER5 software (Rozen and Skaletsky 2000). The primers for the genes examined are listed in Supplementary Table 1. The PCR and detection were executed as described previously (Feng et al. 2013). The 2^−ΔΔCT^ method was used to analyze the relative transcript levels, and the experiments were conducted in triplicate.

### Statistical Analysis

Data were processed with SPSS software 11.5 (IBM Corp., Armonk, NY, USA) to detect differences. The mean values and standard errors in the figures represent data from three experimental replicates. The *t-*test was conducted for normally distributed data. Two-way analysis of variance with two factors (temperature and treatment) was conducted to compare the differences with a LSD test at *P* < 0.05.

## Results

### Response of the Rice Plants to Different Temperatures

**Fig. 1:**
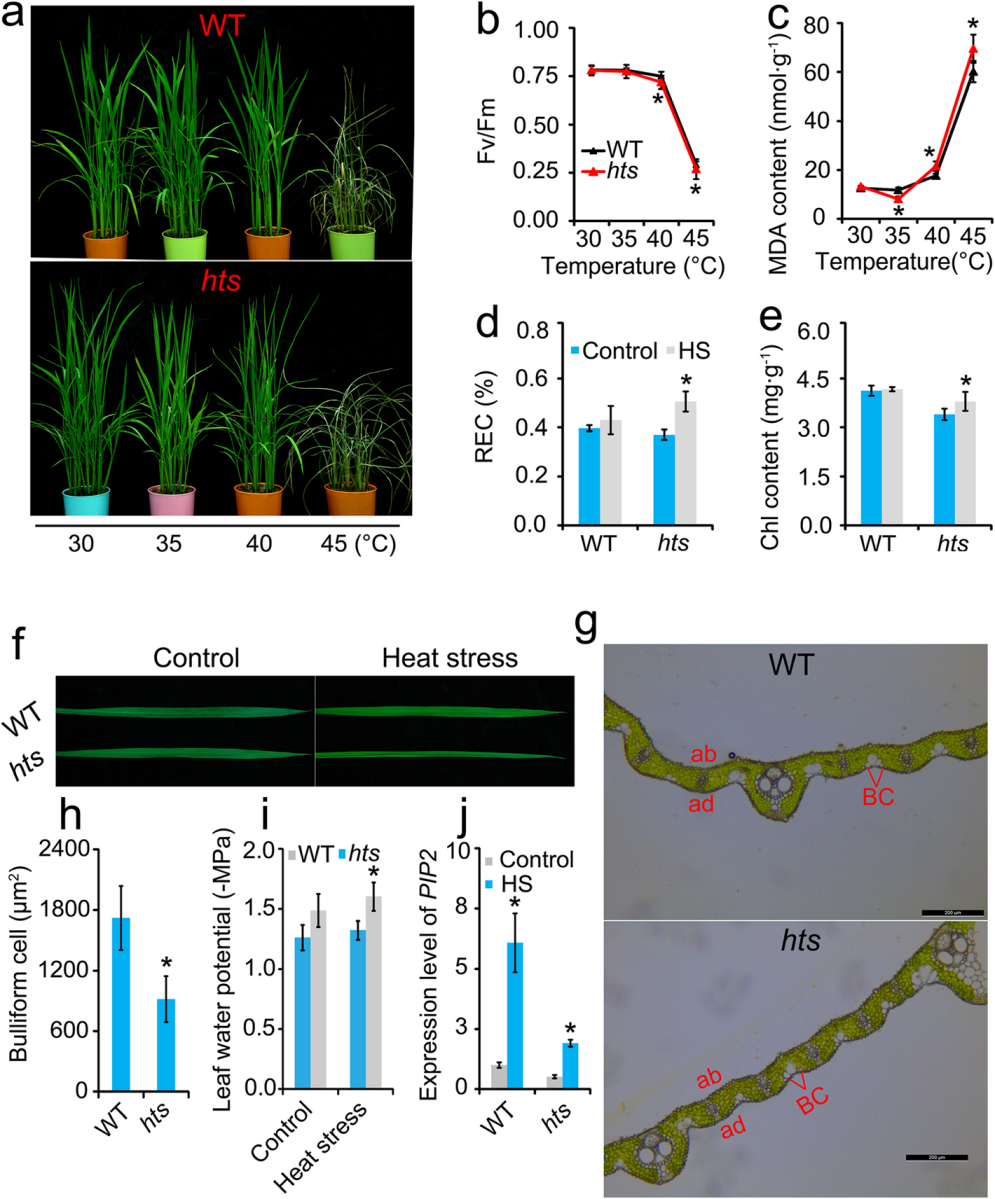
Responses of rice plants to different temperatures under heat stress conditions. **a** Photographs of WT and *hts* plants under different temperature conditions; **b** Fv/Fm; **c**, MDA; **d**, Relative ion leakage; **e**, Chlorophyll content. **f**, Photographs of leaf morphology under control and heat stress conditions; **g**, Photographs of bullioform cell in leaf of WT and *hts* plants under control condition. ‘ad’ stands for adaxial and ‘ab’ for abaxial, ‘BC’ for bulliform cells; h, Mean value of area of bulliform cell; i, Relative leaf water potential; j, Expression levels of *PIP2*. Vertical bars denote standard deviations (*n* = 3). A *t*-test was conducted to compare the difference between control and heat stress within a cultivar on the same day. * denotes *P* < 0.05

To investigate the response of rice plants to different temperature stresses, the WT and *hts* plants were subjected to temperatures of 30 °C, 35 °C, and 40 °C for 72 h, and the plants were subjected to 45 °C heat stress for 24 h (Fig. 1a). Temperatures of 30 °C and 35 °C caused little injury to either plant, but obvious damage was observed in the 40 °C and 45 °C treatments, particularly in the *hts* plants. Accordingly, no obvious difference in Fv/Fm was detected between the WT and *hts* plants under the 30 °C and 35 °C conditions (Fig. 1b). However, the Fv/Fm values of the *hts* plants were significantly lower than those of WT plants under temperatures of 40 °C and 45 °C. In contrast, the MDA content in the leaves of *hts* plants was higher than that of WT plants under the 40 °C and 45 °C treatments (Fig. 1c). No marked differences were detected in REC or chlorophyll content between the control and the 40 °C heat stressed WT plants, while a remarkable increase was found in *hts* plants under heat stress compared with the control (Fig. 1d and e).

The leaves in both rice genotypes appeared to be flat under the control condition of 30 °C (Fig. 1f). Semi-rolled leaves were present on *hts* plants under heat stress of 40 °C, while the leaves of WT plants remained flat. Interestingly, the area of bulliform cells in WT plants was significantly larger than that of *hts* plants under natural conditions (Fig. 1g and h). However, the leaf water potential was higher in WT than that in *hts* plants, particularly under heat stress (Fig. 1i). Aquaporins can maintain water balance in plants under abiotic conditions, and the *PIP2* gene has been reported to function in this process (Kaldenhoff et al. 2008; Israel et al. 2019; Zhang et al. 2019). Similarly, a greater increase in expression levels of *PIP2* were found in WT than that in *hts* plants under heat stress compared with their respective controls (Fig. 1j).

### Effect of Heat Stress on Dry Matter Weight Accumulation, Carbohydrate Content, and Metabolism of Rice Plants

**Fig. 2:**
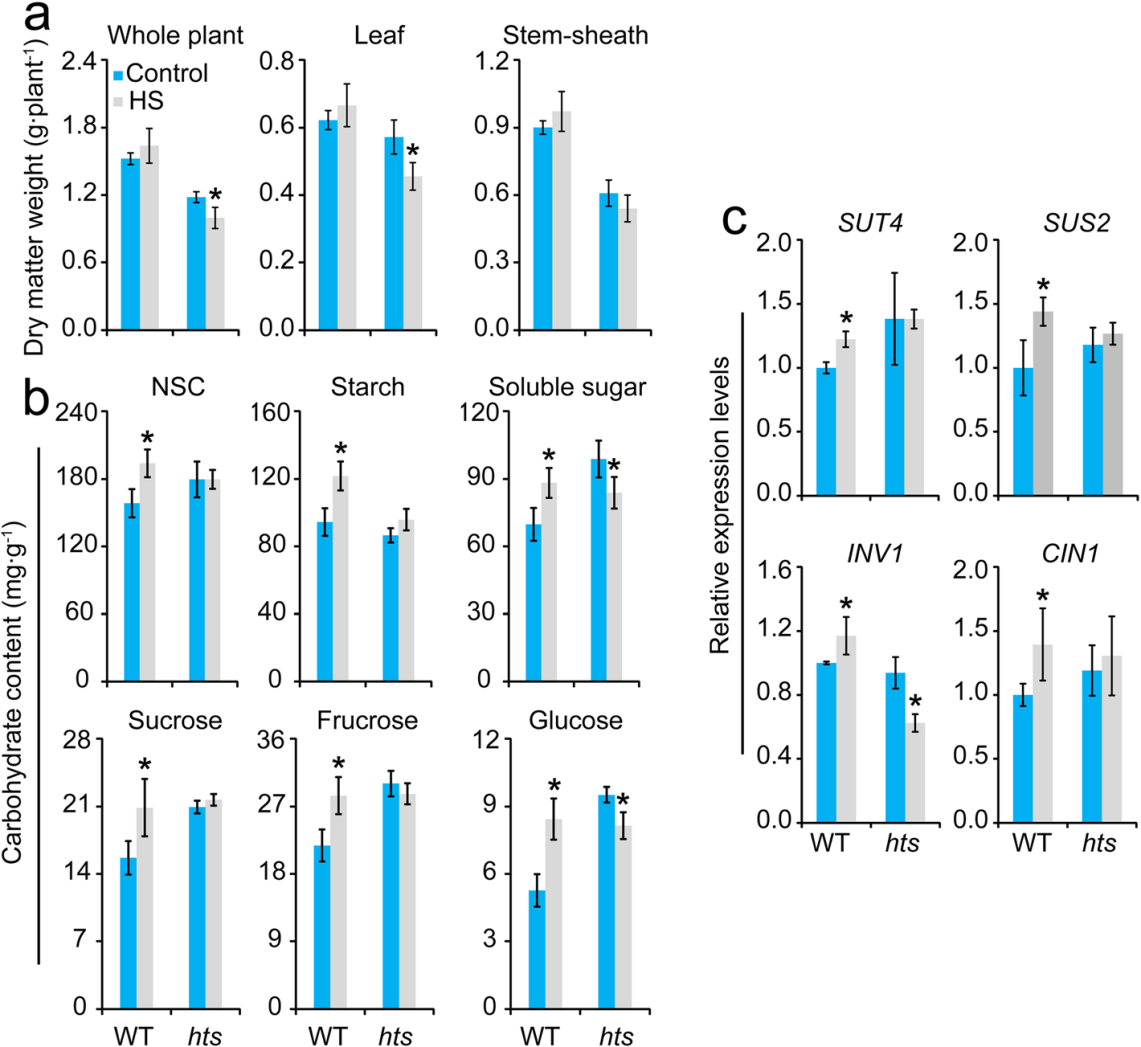
Effect of heat stress on assimilate accumulation and distribution in rice plants under heat stress. **a**, Dry matter weight; **b**, Carbohydrate; **c**, Expression levels of *SUT4*, *SUS2*, *CIN1*, and *INV1*. Vertical bars denote standard deviations (*n* = 3). A *t*-test was conducted to compare the difference between the control and heat stressed groups within a cultivar on the same day. * denotes *P* < 0.05

Sugar allocation and metabolism are important for growth and development of rice plants regardless of the conditions, but they are always disturbed by heat stress (Chen et al. 2019; Islam et al. 2019). Thus, we determined the dry matter weight, carbohydrate content, and genes associated with sucrose transport and metabolism to investigate the changes in sugar transportation and metabolism between WT and *hts* plants in response to heat stress (Fig. 2). No difference was detected in dry matter weight of the whole plant between the control and heat stressed groups of WT plants (Fig. 2a). However, a notable reduction was found in *hts* plants under heat stress compared with the control. Similarly, no significant difference in leaf dry matter weight was observed between the control and heat stressed WT plants, while a remarkable decrease was shown in *hts* plants. However, these results were not found in sheath-stem, where no obvious difference was detected between the control and heat stressed groups in either genotype.

Carbohydrate contents, including non-structural carbohydrates (NSC), starch, soluble sugar, sucrose, glucose, and fructose clearly increased in WT plants under heat stress, compared with their respective controls, whereas such an effect was not observed in *hts* plants (Fig. 2b). No differences in contents of NSC, starch, sucrose or fructose were detected between the control and heat stress in *hts* plants. Additionally, soluble sugar and glucose contents decreased significantly in response to heat stress when compared with the control.

The *SUT4*, *SUS2*, *INV1*, and *CIN1* genes are responsible for sucrose transport and metabolism. As shown in Fig. 2c, the expression levels of *SUT4*, *SUS2*, *INV1*, and *CIN1* increased obviously in WT plants under heat stress compared with the control, while no differences were observed in *hts* plants except for *INV1*, which decreased in response to heat stress.

### Effect of Heat Stress on Leaf Tissue Temperatures in Rice Plants

**Fig. 3:**
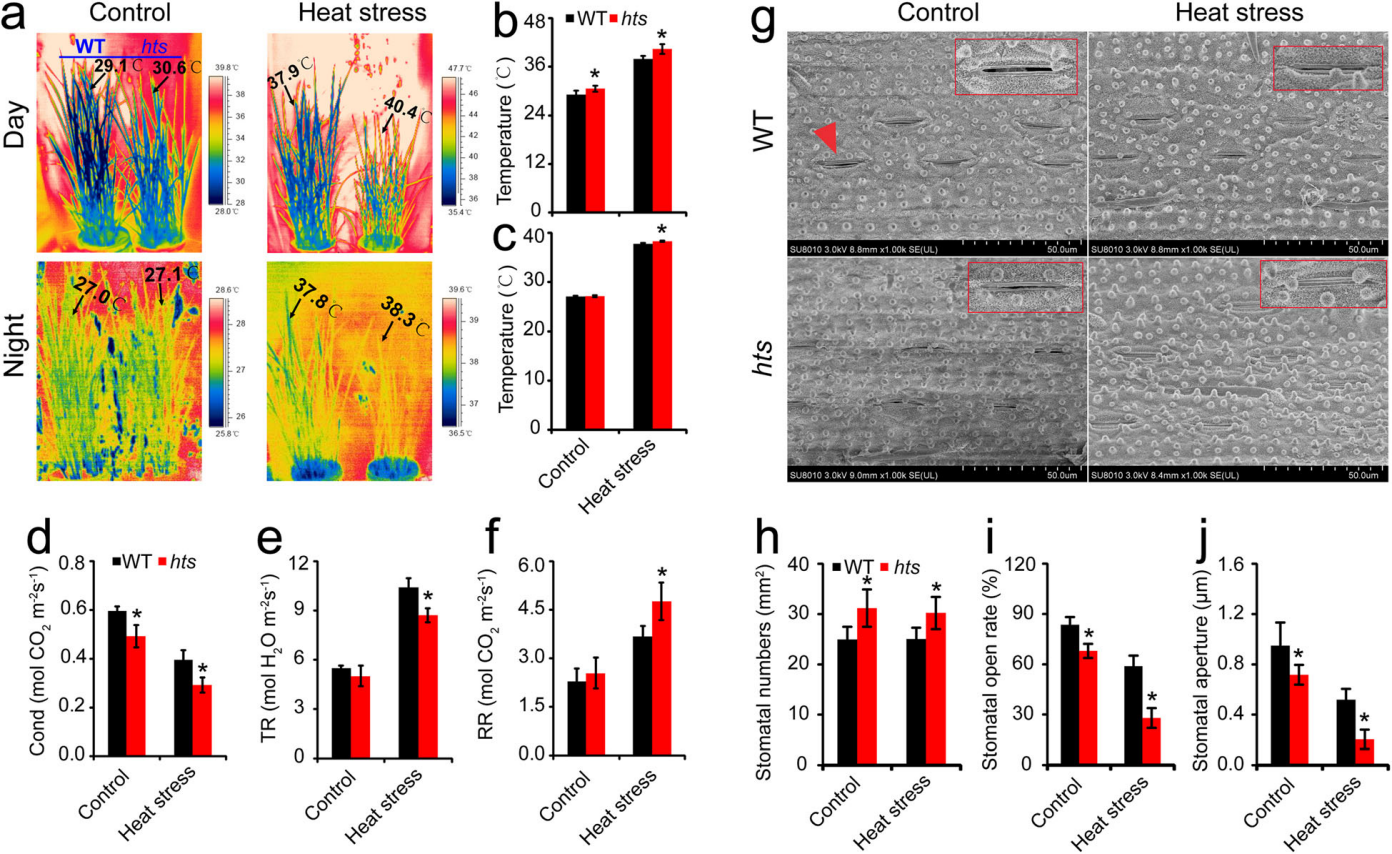
Effect of heat stress on leaf tissue temperature in rice plants. **a**, Thermal images of rice plants under control and heat stress during the day and night; **b** and **c**, Average leaf temperature values at day and night, respectively; **d** and **e**, Stomatal conductance (Cond) and transpiration rates (TR) during the day; **f**, Respiration rate (RR) of leaves at night. **g**, Leaf morphology under control and heats tress, respectively; **h**, Numbers of stomata in leaves; **i**, Stomatal opening rate of leaves; j, Stomatal aperture of leaves. Red triangle indicates the stomata; Red rectangle indicates that the photographs of stomata were taken at 2000-folds. Vertical bars denote standard deviations (*n* = 3). A *t*-test was conducted to compare the difference between the control and heat stress within a cultivar on the same day. * denotes *P* < 0.05

The degree of damage is always associated with leaf tissue temperature in rice plants under heat stress (Fu et al. 2016; Wu et al. 2019). Higher tissue temperature not only directly damages rice plants but also consumes more carbohydrates through respiration, which results in energy deficits and heat damage (Baena-González and Sheen 2008; Islam et al. 2018). As shown in Fig. 3, the leaf temperatures of WT plants were lower during the day time than leaves in *hts* plants, and the differences were about 1.5 °C and 2.5 °C under the control and heat stress conditions, respectively (Fig. 3a and b). No difference in leaf temperature was detected at night between the WT and *hts* plants under the control condition (Fig. 3c). However, the leaf temperature in WT plants was lower by 0.5 °C than that of *hts* plants under heat stress.

As the main factors contributing to leaf temperature, stomatal conductance and transpiration rates were determined during the day. Stomatal conductance of leaves in WT plants was significantly higher than that of *hts* plants (Fig. 3d). No obvious difference was detected in the transpiration rate between the two plants under control conditions, whereas the difference was significant under the heat stressed condition (Fig. 3e). No difference in respiration was detected between WT and *hts* plants under the control condition, but the respiration rate of *hts* plant leaves was significantly higher than that of WT plant leaves under the heat stressed condition (Fig. 3f).

The stomata numbers, stomatal opening rate, and aperture of the leaves were also determined under heat stress during the day (Fig. 3g). Based on the photographs, we found higher numbers of stomata in *hts* than WT plants (Fig. 3h). In contrast, the stomatal opening rate and stomatal aperture in WT plants were obviously higher than those of *hts* plants, particularly under heat stress (Fig. 3i and j).

### Effect of Heat Stress on ABA, H_2_O_2_, and Heat Shock Proteins of Leaves in Rice Plants

**Fig. 4:**
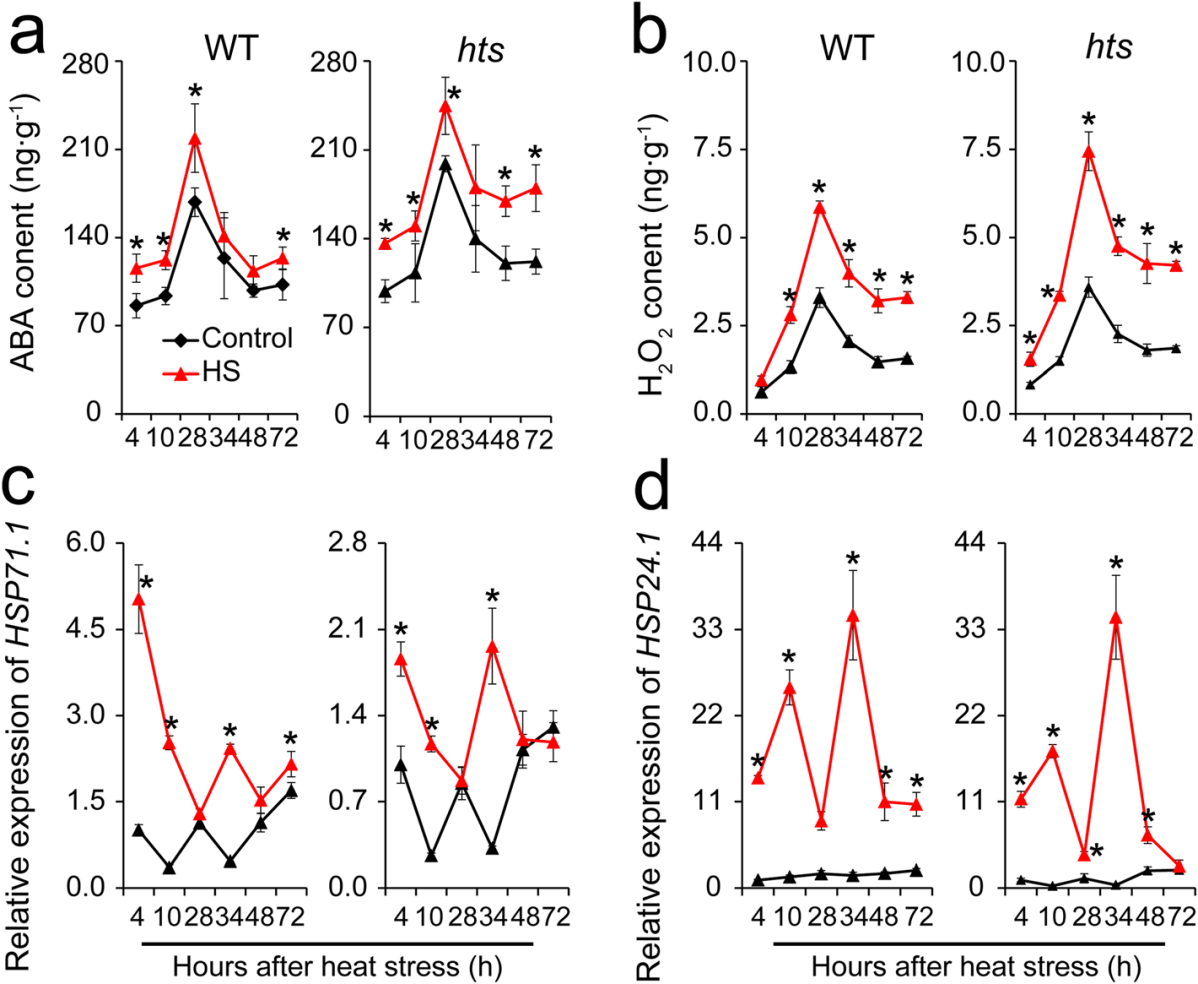
Changes in of ABA, H_2_O_2_, and heat shock proteins in leaves of rice plants under heat stress. **a**, ABA content; **b**, H_2_O_2_ content; **c**, Relative expression level of *HSP71.1*; **d**, Relative expression level of *HSP24.1*. Vertical bars denote standard deviations (*n* = 3). A *t*-test was conducted to compare the differences between control and heat stress within a cultivar on the same day. * denotes *P* < 0.05

ABA and H_2_O_2_ not only play critical roles in stomatal aperture and transpiration capacity of leaves (Feng et al. 2020; Islam et al. 2018), but also induce the accumulation of HSPs (Li et al. 2014). ABA and H_2_O_2_ contents as well as the expression levels of the *HSP71.1* and *HSP24.1* genes were determined to reveal the relationship among ABA, H_2_O_2_, and HSPs in these two rice plants under heat stress. Similar changes in ABA and H_2_O_2_ were detected in the two plants, in which higher ABA and H_2_O_2_ contents were found in *hts* plants than in WT plants regardless of the conditions (Fig. 4a and b). Additionally, greater increases in ABA and H_2_O_2_ contents were found in *hts* plants than WT plants under heat stress compared with their respective controls, particularly 34–72 h after the heat stress. Notably, a greater increase in *HSP71.1* and *HSP24.1* expression levels was detected in WT than *hts* plants under heat stress, particularly 48–72 h after the heat stress (Fig. 4c and d).

### Effect of Heat Stress on Energy Homeostasis of Leaves in Rice Plants

**Fig. 5:**
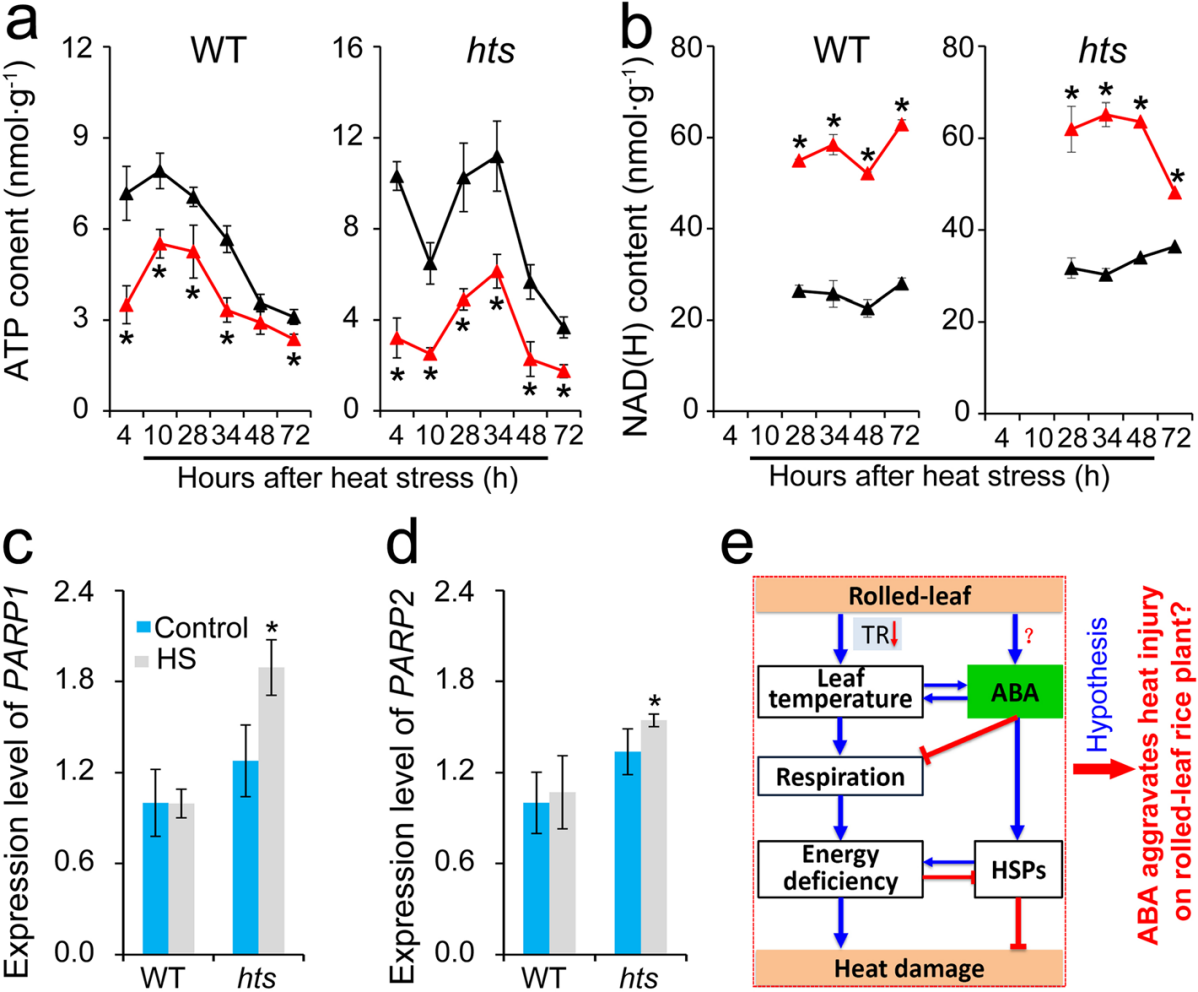
Effect of heat stress on energy homeostasis in leaves of rice plants. **a**, ATP content*;***b***,* NAD (H) content; **c**, Relative expression level of *PARP1*; **d**, Relative expression level of *PARP2*; **e**, The possible function of ABA in rice plants with semi-rolled leaves under heat stress. Significantly higher tissue temperatures occurred in the rolled leaf rice plants (*hts*) under heat stress, which was mainly ascribed to lower heat dissipation ability. At the same time, ABA content increased in the rolled leaf plants, which, in turn, increased leaf temperature by reducing the stomatal conductance and transpiration rates. Such an effect could increase the respiration rate of leaves. Therefore, more carbohydrates were consumed, resulting in an energy deficit under heat stress, which caused damage to the rice plants with rolled leaves. Therefore, we inferred that higher ABA content accelerated heat injury in rice plants with rolled leaves. Vertical bars denote standard deviations (*n* = 3). A *t*-test was conducted to compare the difference between the control and heat stressed groups within a cultivar on the same day. * denotes *P* < 0.05

HSPs provide protection against heat stress in plants, but their accumulation is a high cost energy process (Grenert et al. 1997). ATP and NAD (H) contents as well as the expression levels of PARP genes were determined to investigate the effect of heat stress on energy production and consumption in plants (Fig. 5). ATP content decreased markedly in response to heat stress compared with the control in both genotypes, where a greater decrease was found in *hts* than WT plants during the entire stress period (Fig. 5a). In contrast, a sharp increase in NAD (H) content of leaves was observed in both rice plants under heat stress, and a greater increase was found in WT than *hts* plants, particularly 72 h after heat stress (Fig. 5b). PARP is related to energy consumption in plants under abiotic stress (Tiwari et al. 2002; De Block et al. 2011), and no significant differences were observed in *PARP1* or *PARP2* expression between the control and heat-stressed groups of WT plants, while greater increases were detected in *hts* plants under heat stress (Fig. 5c and d).

### The Function of ABA in Heat Tolerance of *hts* Plants under Heat Stress

According to the above results, ABA could negatively regulate heat acclimation in *hts* plants with rolled leaves (Fig. 5e). A higher leaf temperature was always observed in *hts* plants with semi-rolled leaves under the heat stressed condition, which was mainly due to their lower heat dissipation ability and enhanced ABA content in leaves, which, in turn, increased leaf temperature by inducing stomatal closure to reduce the transpiration rate. Higher respiration rates of leaves caused by higher tissue temperatures consume more carbohydrates, resulting in an energy deficit under heat stress, which could impair the ability of the plants to overcome peak stress. Therefore, we hypothesized ABA adversely affected heat acclimation of rice plants with semi-rolled leaves to heat stress.

### Effect of ABA on Tissue Temperature of Rice Plant Leaves under Heat Stress

**Fig. 6:**
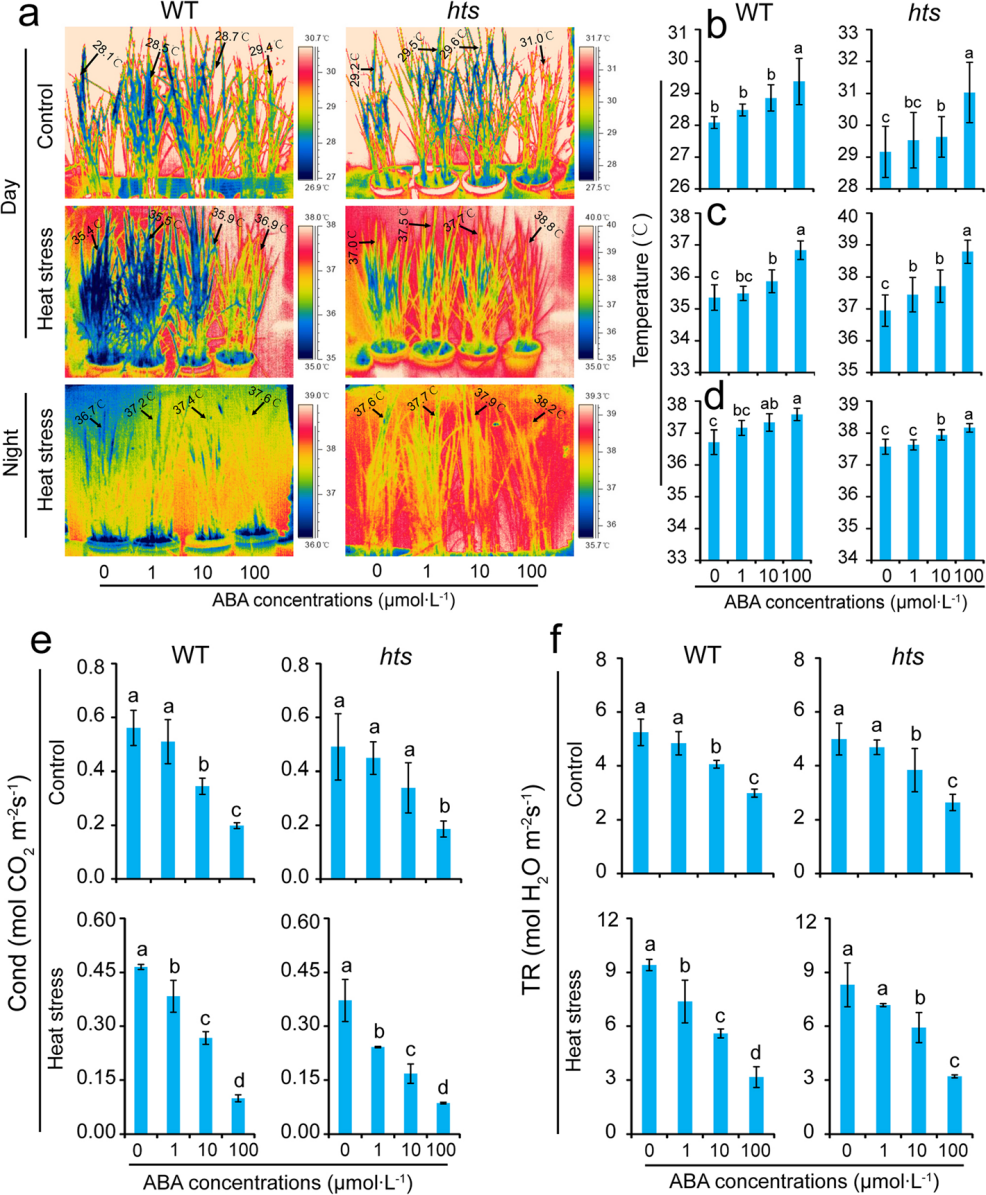
Changes in tissue temperatures, stomatal conductance, and transpiration rates of rice plant leaves caused by ABA under heat stress. **a**, Thermal images of rice plants under the control and heat stress treatments during the day and night; **b**, Average leaf tissue temperature values at day under control conditions; **c**, Average leaf tissue temperature values at day under heat stress; **d**, Average leaf tissue temperature values at night under heat stress; **e**, Stomatal conductance (Cond); **f**, Transpiration rate (TR). Vertical bars denote standard deviations (*n* = 10 or 3). Different letters indicate significant differences among ABA treatments under the control and heat stress conditions within a genotype by two-way analysis of variance for two factors (temperature and treatment) (*P* < 0.05)

Different ABA concentrations were sprayed onto rice plants to reveal the role of ABA in heat tolerance between WT and *hts* plants under heat stress. Leaf temperatures increased as the ABA concentration was increased in both rice plants regardless of the conditions (Fig. 6a and b). Under heat stress, the leaf temperatures of WT plants increased by 0.1 °C, 0.5 °C, and 1.5 °C in the 1 μmol·L^− 1^, 10 μmol·L^− 1^, and 100 μmol·L^− 1^ ABA treatments, respectively compared with that of the 0 μmol·L^− 1^ ABA treatment, while those of *hts* plants were 0.5 °C, 0.7 °C, and 1.8 °C, respectively (Fig. 6c). At night, the leaf temperatures in WT plants sprayed with 0 μmol·L^− 1^, 1 μmol·L^− 1^, 10 μmol·L^− 1^, and 100 μmol·L^− 1^ ABA were about 36.7 °C, 37.2 °C, 37.4 °C, and 37.6 °C under heat stress, respectively, while in *hts* plants the temperatures were 37.6 °C, 37.7 °C, 37.9 °C, and 38.2 °C (Fig. 6d). Stomatal conductance and transpiration rates decreased significantly in response to ABA in both genotypes, particularly in plants treated with 100 μmol·L^− 1^ ABA (Fig. 6e and f).

### Effect of ABA on Fv/Fm, Sugar Transport, and Metabolism of Rice Plant Leaves under Heat Stress

**Fig. 7:**
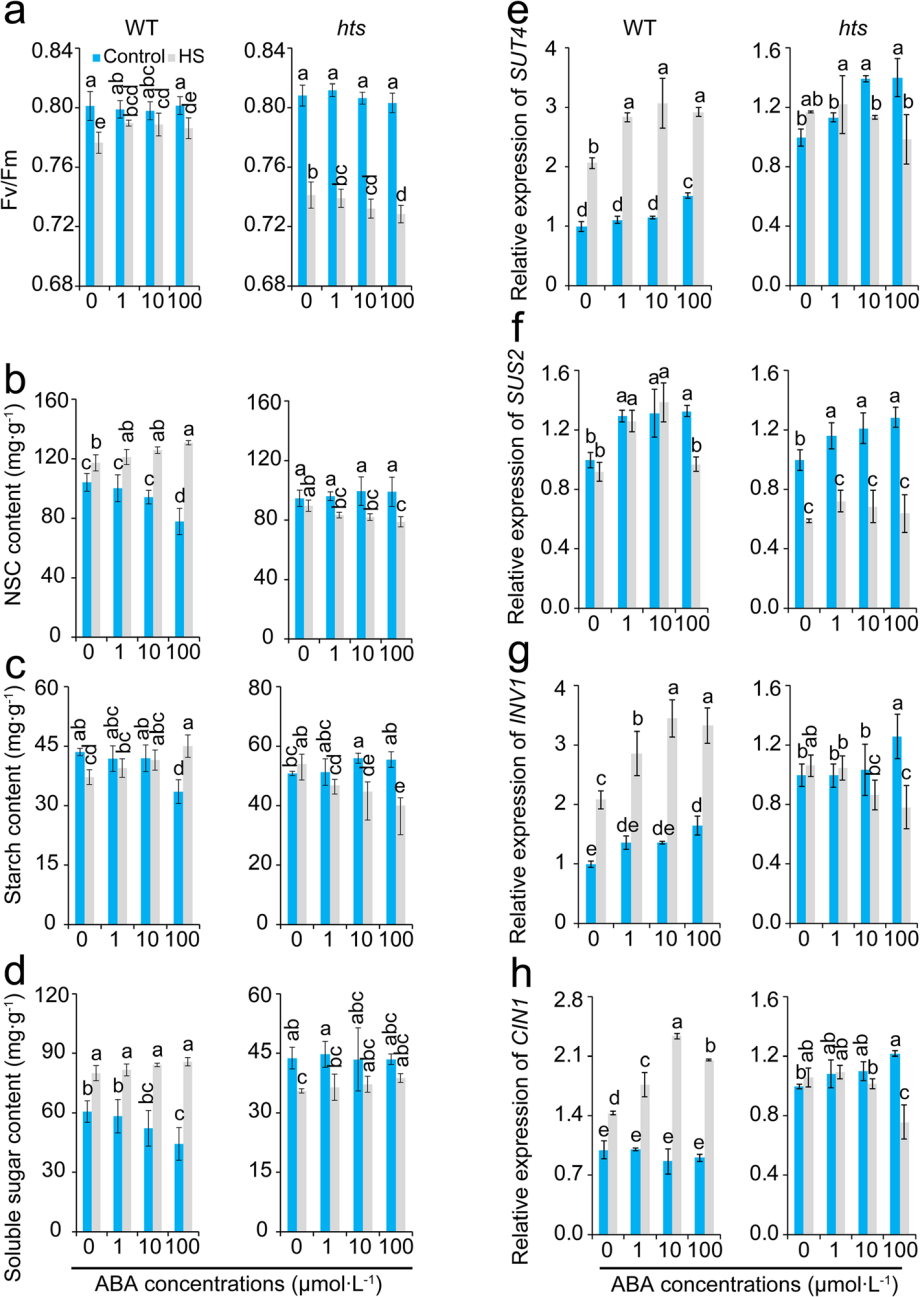
Effect of ABA on Fv/Fm, carbohydrate content and metabolism of leaves in rice plants under heat stress. **a**, Fv/Fm; **b**, NSC content; **c**, starch content; **d**, soluble sugar content; **e**, Relative expression level of *SUT4*; **f**, Relative expression level of *SUS2*; **g**, Relative expression level of *INV1*; **h**, Relative expression level of *CIN1*. Vertical bars denote standard deviations (*n* = 3). Different letters indicate a significant difference among ABA treatments under the control and heat-stressed conditions within a genotype by two-way analysis of variance for two factors (temperature and treatment) (*P* < 0.05)

The Fv/Fm is reported to be susceptible to heat stress and is commonly used to evaluate heat tolerance in plants (Poudyal et al. 2019). As shown in Fig. 7a, the Fv/Fm value decreased significantly in response to heat stress. Notably, the decrease in Fv/Fm by heat stress was reversed by ABA in WT plants. In contrast, the Fv/Fm values in *hts* plants decreased as the ABA concentration was increased under heat stress, particularly the 10 μmol·L^− 1^ and 100 μmol·L^− 1^ ABA treatments.

The contents of NSC, starch, and soluble sugar in the leaves of WT plants decreased with the increase in ABA concentration under control conditions (Fig. 7b, c and d). In contrast, there was almost no difference in contents of NSC, starch, or soluble sugar among these treatments of *hts* plants under the control conditions. Interestingly, in WT plants, the contents of NSC, starch, and soluble sugar increased as the ABA concentrations was increased under heat stress, while they decreased in *hts* plants, except for soluble sugar. The expression levels of genes related the sucrose transport and metabolism, such as *SUT4*, *SUS2*, *INV1*, and *CIN1* increased in response to ABA except for *CIN1* in WT plants under the control conditions (Fig. 7e-h). Interestingly, these genes in WT plants were induced by heat stress or ABA, while they decreased significantly in *hts* plants in response to ABA compared with their respective controls.

### Effect of ABA on Energy Production and Consumption of Leaves in Rice Plants under Heat Stress

**Fig. 8:**
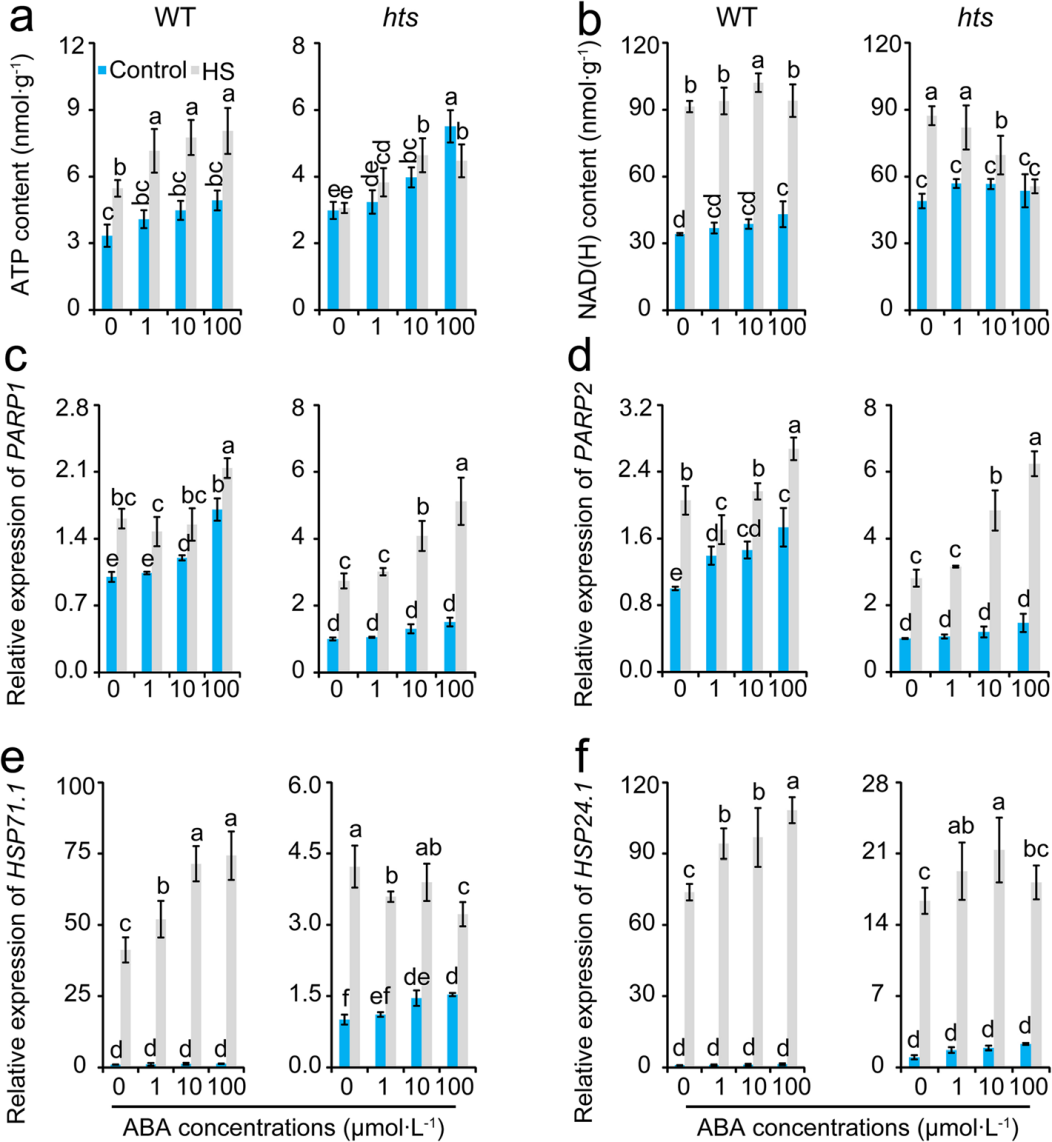
Changes in ATP and NAD (H) contents as well as the expression levels of *HSP* genes in leaves of rice plants in response to ABA under heat stress. **a**, ATP content; **b**, NAD (H) content; **c**, Relative expression level of *PARP1*; **d**, Relative expression level of *PARP2*; **e**, Relative expression level of *HSP71.1*; **f**, Relative expression level of *HSP24.1*. Vertical bars denote standard deviations (*n* = 3). Different letters indicate a significant difference among the ABA treatments under the control and heat-stressed conditions within a genotype by two-way analysis of variance for two factors (temperature and treatment) (*P* < 0.05)

Regardless of the conditions, ATP content increased with increased ABA concentration (Fig. 8a). Interestingly, ATP content in WT plants increased significantly in response to heat stress, whereas no difference was detected between the control and heat stress in *hts* plants except for the 100 μmol·L^− 1^ ABA treatment. No significant differences in NAD (H) were detected among the ABA treatments under the control condition except for the 100 μmol·L^− 1^ ABA treatment in WT plants, which was significantly higher than that of the 0 μmol·L^− 1^ ABA treatment (Fig. 8b). A sharp increase in NAD (H) content was detected in WT plants under heat stress, especially the 10 μmol·L^− 1^ ABA treatment, while NAD (H) content decreased in *hts* plants as ABA concentration was increased. The *PARP* genes were activated by heat stress in both genotypes, but different responses to ABA were found between the WT and *hts* plants (Fig. 8c and d). Compared with their respective controls, the lower increase in the expression levels of *PARP1* and *PARP2* were detected in WT plants than *hts* plants under heat stress, particularly in the 100 μmol·L^− 1^ ABA treatment.

### Effect of ABA on the Relative Expression Levels of *HSP* Genes in Leaves under Heat Stress

Under the control condition, the expression levels of *HSP71.1* and *HSP24.1* increased as ABA concentration was increased, while no difference was detected among the ABA treatments except for *HSP71.1* in *hts* plants (Fig. 8e and f). The expression levels of *HSP71.1* and *HSP24.1* in WT plants increased significantly under heat stress in response to ABA. Similarly, heat stress induced expression of *HSP71.1* and *HSP24.1* in *hts* plants, whereas the increases in expression levels decreased as ABA concentration was increased under heat stress. Additionally, a greater increase in *HSP71.1* and *HSP24.1* expression levels were found in WT than *hts* plants under heat stress compared with their respective controls, particularly the 100 μmol·L^− 1^ ABA treatment.

## Discussion

### Contribution of the Semi-Rolling Trait Leaf to Heat Resistance in Rice Plants

The present results indicate that more damage was caused by heat stress in *hts* plants with semi-rolled leaves than in WT plants at 40 °C or 45 °C (Fig. 1), which was consistent with our previous results (Zhang et al. 2018). The semi-rolled trait leaf may have contributed to lower heat tolerance in *hts* plants (Fig. 1a and b). This finding was inconsistent with previous results that the rolled leaf trait confers resistance to plants against drought and heat stress by reducing transpiration and water loss from plants to improve water use efficiency (O’Toole and Cruz 1980; Sarieva et al. 2010; Buitrago et al. 2016; Zhang et al. 2018; Cal et al. 2019). However, this function of rolled leaves enhances leaf temperatures, resulting in more damage to the rice plants under a heat stress condition (Fig. 3). Heat stress causes more damage to spikelets than leaves of rice plants mainly because of the higher tissue temperatures in spikelets than leaves (Zhang et al. 2016).

Indeed, the rolled leaf trait in *hts* plants was only found under the heat stress condition (Fig. 1f). According to the present results (Fig. 1 and Fig. S1), bulliform cells might not be involved in the process of leaf rolling in *hts* plants under heat stress (Li et al. 2010). Notably, markedly greater decreases in leaf water potential were found in *hts* than WT plants under heat stress (Fig. 1i), which could explain the changes in leaf morphology between the two genotypes (Fig. 1a and f). We inferred that aquaporins might play critical roles in this process, as they could maintain water balance in plants under abiotic stress by reducing water loss from leaves and enhancing water uptake at the roots (Moshelion et al. 2015; Maurel et al. 2016; McGaughey et al. 2018; Chen et al. 2018; Franzini et al. 2019; Quiroga et al. 2019). The present results indicate that a significant increase in *OsPIP2* expression level was detected in leaves of WT plants than that in *hts* plants under the heat stress condition (Fig. 1j). This was consistent with the result that aquaporins are induced in strawberry plants to acquire systemic thermotolerance (Christou et al. 2014). However, this gene could be induced by ABA in either plant, regardless of the conditions, while the leaf water potential in both rice plants decreased as ABA concentration was increased (Fig. S5). This finding suggests that *OsPIP2* might also be induced in roots to enhance water uptake under heat stress.

Aquaporins may function in heat tolerance of these two plants. However, the data are insufficient to confirm this hypothesis, and thus the mechanism underlying heat tolerance between these two rice plants remains unclear. Many genes, including *TT1* and *ERECTA* have been identified to confer heat tolerance in rice, *Arabidopsis*, tomato, and other species (Li et al. 2015; Shen et al. 2015; Jacob et al. 2017; Brzezinka et al. 2019; Rytz et al. 2018). These genes provide protection against heat stress in plants or enhance their biomass under high temperature conditions. However, the genes controlling the rolled-leaf of *hts* plants and heat tolerance in these two rice plant genotypes have been not cloned. Therefore, we are planning to clone the genes through an RFLP map from a DH population. Thereafter, transcriptome, proteome, metabolome, and yeast-two-hybrid assays will be used to investigate the functions of the genes in heat tolerance as well as their response to ABA under heat stress.

### The Negative Role of ABA in Conferring Heat Tolerance in Rolling Leaf Rice Plants

In this study, higher ABA and H_2_O_2_ levels in leaves were shown in *hts* than in WT plants under heat stress (Fig. 4a and b), suggesting that ABA was a negative regulator of *hts* heat tolerance. This hypothesis was confirmed by the present results, that the Fv/Fm values of *hts* plant leaves decreased as exogenous ABA concentration was increased (Fig. 7a). Clearly, this finding was inconsistent with the result that ABA confers heat tolerance in plants (Li et al. 2014; Suzuki et al. 2016; Cho et al. 2018; Hu et al. 2018; Islam et al. 2019). These contradictory results depended on the morphology of the leaves under heat stress, as heat tolerance was enhanced in WT plants by exogenous ABA (Fig. 7a). Importantly, similar results were also found in the IR64 (a wild type), rolled leaf mutant *RL241*, and *RL291* plants treated with ABA under heat stress (Figs. S3 and S4). Therefore, the increase in ABA level in *hts* plants was the result of heat damage, rather than a protective strategy against heat stress.

The negative role of ABA in the heat response of *hts* plants might be related with an energy deficit. It has been well documented that accumulation of HSPs is important to tolerate heat stress (Islam et al. 2019), which can be induced by ABA, H_2_O_2_, and high temperatures (Li et al. 2014). However, greater increases in the relative expression levels of *HSP71.1* and *HSP24.1* were found in WT than *hts* plants under heat stress (Fig. 4c and d), which was inconsistent with the previous results (Li et al. 2014; Isalm et al. 2019). Indeed, the accumulation of HSPs is a high energy cost process that can be inhibited by the energy deficit caused by heat stress. The greater decreases in dry matter weight, carbohydrates, ATP, and NAD (H) were showed in *hts* than WT plants under heat stress (Figs. 2, 4 and 5), suggesting higher energy costs were showed in *hts* plants with semi-rolled leaves under heat stress, which might be mainly due to higher leaf temperatures and respiration (Fig. 3). This hypothesis was confirmed by the present results, that the carbohydrate, ATP, and NAD (H) contents decreased in *hts* plants as the ABA concentration increased under heat stress (Figs. 7 and 8). Accordingly, the increased expression levels of *HSP71.1* and *HSP24.1* decreased significantly in *hts* plants under heat stress as the ABA concentration was increased (Fig. 8e and f). Notably, such effects caused by ABA were not found in WT plants.

Higher energy cost in *hts* plants under heat stress might be related to PARP. It has been reported that silencing *AtPARP1* or *AtPARP2* in *Arabidopsis* enhances plant tolerance to drought, high light, and heat stress (Vanderauwera et al. 2007; Briggs and Bent 2011; Rissel et al. 2017). Plants with reduced PARP activity consume less NAD (H) in stressful environments to improve their energy-use efficiency by reducing over-active mitochondrial respiration and ROS production (Tiwari et al. 2002; De Block et al. 2011). The present results indicate that greater increases in *PARP1* and *PARP2* expression levels were found in *hts* than WT plants under heat stress (Fig. 5c and d). Importantly, the expression levels of the *PARP* genes increased as ABA concentration was increased in both WT and *hts* plants (Fig. 8c and d), suggesting that PARP could be activated by ABA. However, this effect depended on the genotype, which might be due to different leaf tissue temperatures caused by heat stress or ABA (Fig. 6a-d).

## Conclusion

Heat stress caused more damage to *hts* plants with semi-rolled leaves than WT plants with flat leaves, which was mainly ascribed to higher tissue temperatures and respiration rates as well as lower transpiration and stomatal conductance rates of leaves. Higher increases in levels of ABA and H_2_O_2_ were found in *hts* than WT plants under heat stress compared with their respective controls. However, greater increase in contents of dry matter weight, carbohydrates, ATP, and NAD (H) as well as expression levels of *HSP71.1* and *HSP24.1* were observed in WT than *hts* plants. These results suggest that ABA negatively regulates heat tolerance in *hts* plants. This hypothesis was confirmed by the further results that the increases in Fv/Fm, carbohydrates, ATP, and NAD (H) as well as the expression levels of *HSP71.1* and *HSP24.1* in *hts* plants increased in response to exogenous ABA in WT plants, while the increases were reduced in *hts* plants. Therefore, we infer that ABA negatively modulates semi-rolled leaf rice against heat stress by enhancing leaf temperature and reducing transpiration, resulting in an energy deficiency.

## Supplementary information

**Additional file 1: Fig. S1.** Effect of heat stress on the expression levels of genes associated with bulliform cells in leaves of rice plants. a, Relative expression level of *Tid1*; b, Relative expression levels of *ACL1*. Vertical bars denote standard deviations (*n* = 3). A *t*-test was conducted to compare the difference between the control and heat stressed groups within a cultivar on the same day. *denotes *P* < 0.05.

**Additional file 2: Figure S2.** Effect of heat stress on the expression levels of genes associated with aquaporins in leaves of rice plants. a, Relative expression level of *TIP1*; b, Relative expression level of *TIP4*. Vertical bars denote standard deviations (*n* = 3). A *t*-test was conducted to compare the difference between the control and heat stressed groups within a cultivar on the same day. * denotes *P* < 0.05.

**Additional file 3: Figure S3** Leaf mophology of IR64 and its mutant *RL241* under control conditions.

**Additional file 4: Figure S4.** Effect of ABA on tissue temperature and Fv/Fm of leaves in IR64, *RL241* and *RL291* plants under heat stress. a and b, Thermal images of rice plants under the heat stress and control treatments without ABA treatment, respectively; c and d, Thermal images of IR64 and *RL241* plants under the control with ABA treatments; e and f, Thermal images of IR64 and *RL241* plants under heat stress with ABA treatments; g, Leaf temperature of IR64 and *RL241* without ABA treatment; h-j; Leaf temperature of IR64 and *RL241* with ABA treatments; i-n, Fv/Fm of IR64, *RL241* and *RL291*. Vertical bars denote standard deviations (Tissue temperature, *n* = 10; Fv/Fm, *n* = 5). Different letters indicate a significant difference among the ABA treatments under the control and heat-stressed conditions within a genotype by two-way analysis of variance for two factors (temperature and treatment) (*P* < 0.05).

**Additional file 5: Figure S5.** Effect of ABA on expression levels of the *PIP2* gene and water potential of leaves in rice plants under heat stress. a and b, Relative expression of *PIP2*; c and d, Leaf water potential. Vertical bars denote standard deviations (*PIP2*, *n* = 3; Leaf water potential, *n* = 4). Different letters indicate a significant difference among the ABA treatments under the control and heat-stressed conditions within a genotype by two-way analysis of variance for two factors (temperature and treatment) (*P* < 0.05).

**Additional file 6: Table S1.** Primer sequences used in qRT-PCR.

## Data Availability

The datasets supporting the conclusions of this article are included with in the article (and its additional files).

## Abbreviations

ABA: Abscisic acid
Fv/Fm: Maximum fluorescence quantum efficiency
HS: Heat stress
HSP: Heat shock protein
*hts*: *High temperature susceptible*
MDA: Malondialdehyde
NSC: Non-structural carbohydrates
PARP: Poly (ADP-Ribose) Polymerases
WT: wild type
