# Supplementary figures and images for: Abscisic Acid Negatively Modulates Heat Tolerance in Rolled Leaf Rice by Increasing Leaf Temperature and Regulating Energy Homeostasis
Source: Rice (N Y). 2020 Mar 13;13:18. doi: 10.1186/s12284-020-00379-3 (PMC7070142; doi:10.1186/s12284-020-00379-3)

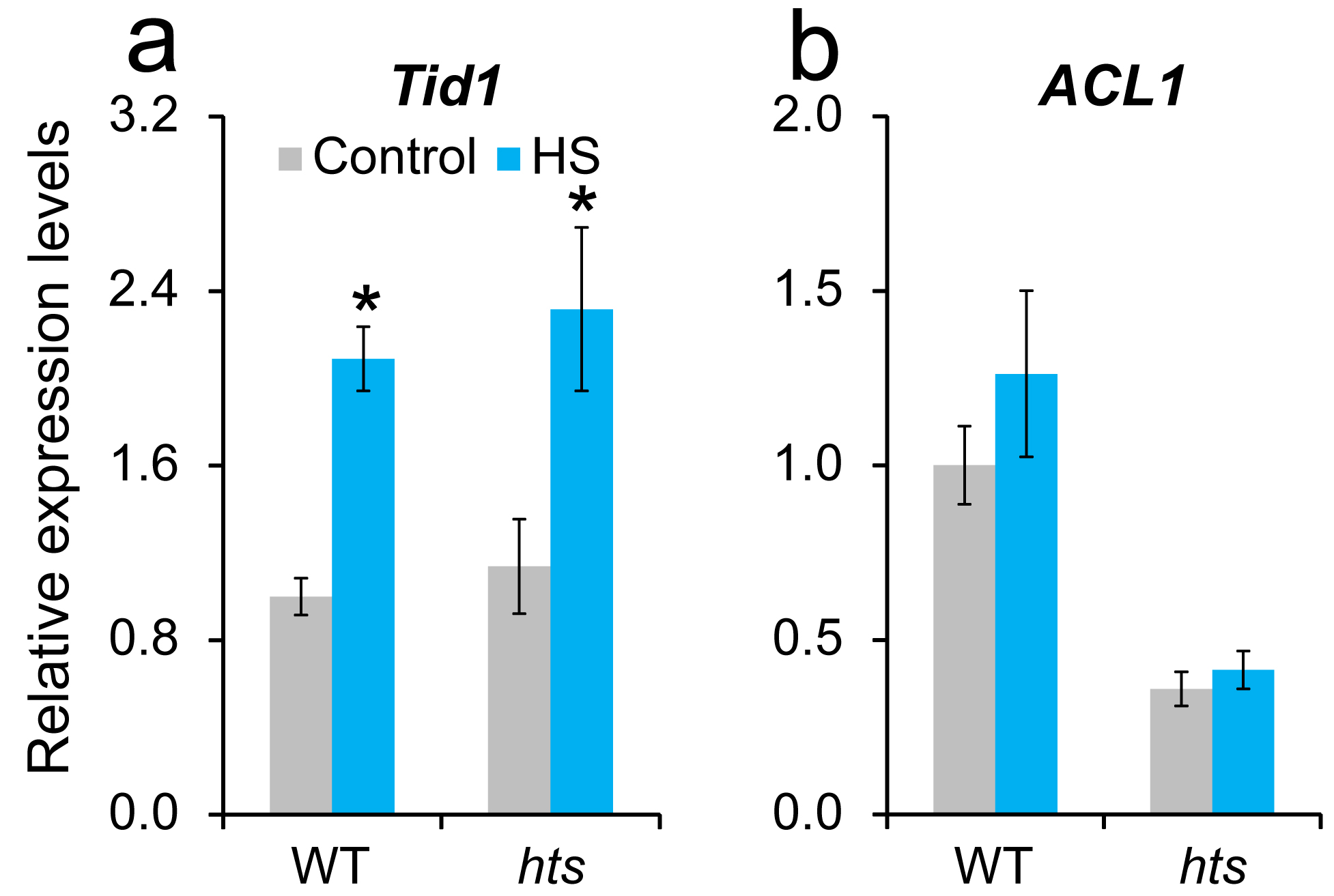

Supplement: Supplementary file 1 — Additional file 1: Fig. S1. Effect of heat stress on the expression levels of genes associated with bulliform cells in leaves of rice plants. a, Relative expression level of Tid1; b, Relative expression levels of ACL1. Vertical bars denote standard deviations (n = 3). A t-test was conducted to compare the difference between the control and heat stressed groups within a cultivar on the same day. *denotes P < 0.05. [file 12284_2020_379_MOESM1_ESM.jpg]

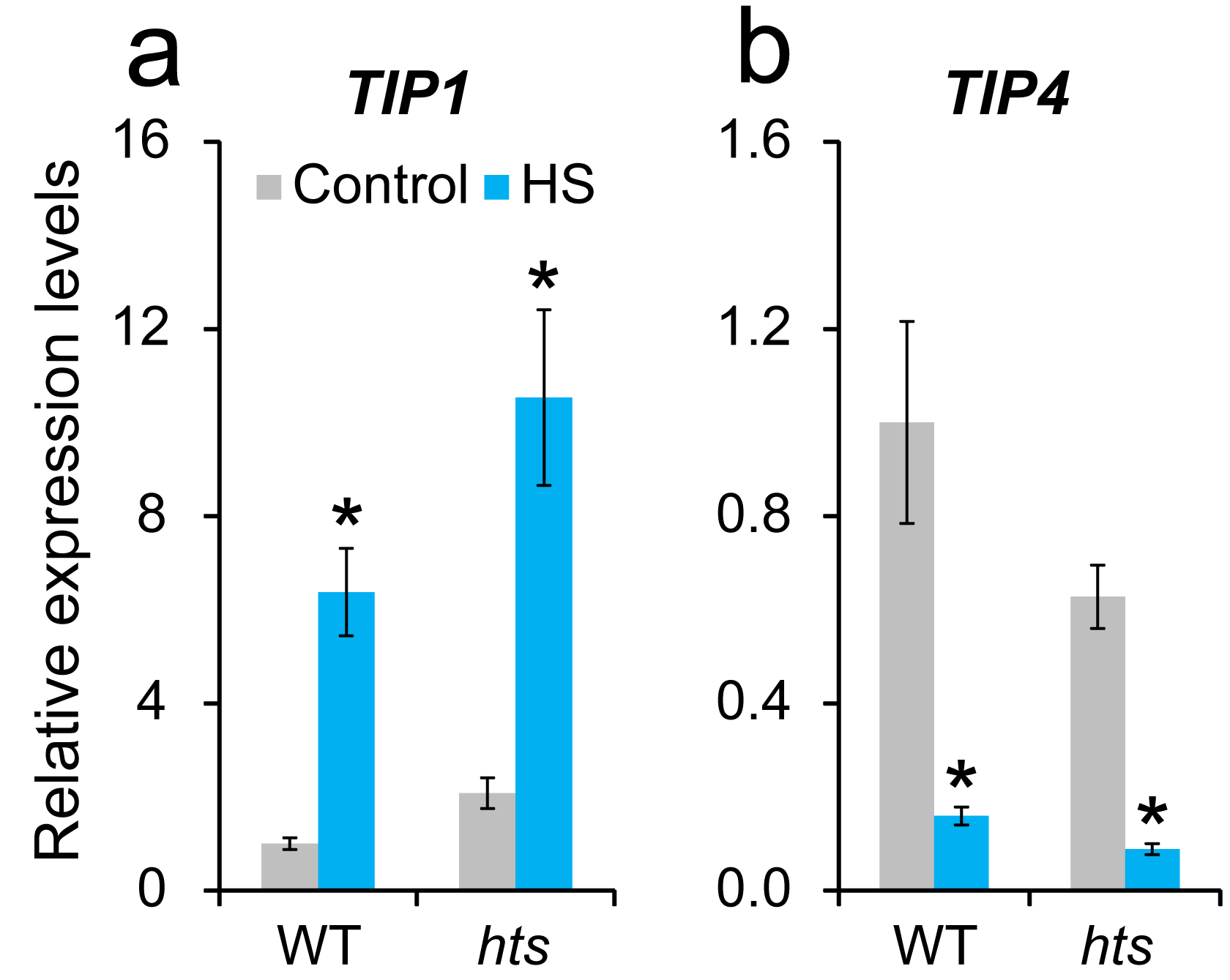

Supplement: Supplementary file 2 — Additional file 2: Figure S2. Effect of heat stress on the expression levels of genes associated with aquaporins in leaves of rice plants. a, Relative expression level of TIP1; b, Relative expression level of TIP4. Vertical bars denote standard deviations (n = 3). A t-test was conducted to compare the difference between the control and heat stressed groups within a cultivar on the same day. * denotes P < 0.05. [file 12284_2020_379_MOESM2_ESM.jpg]

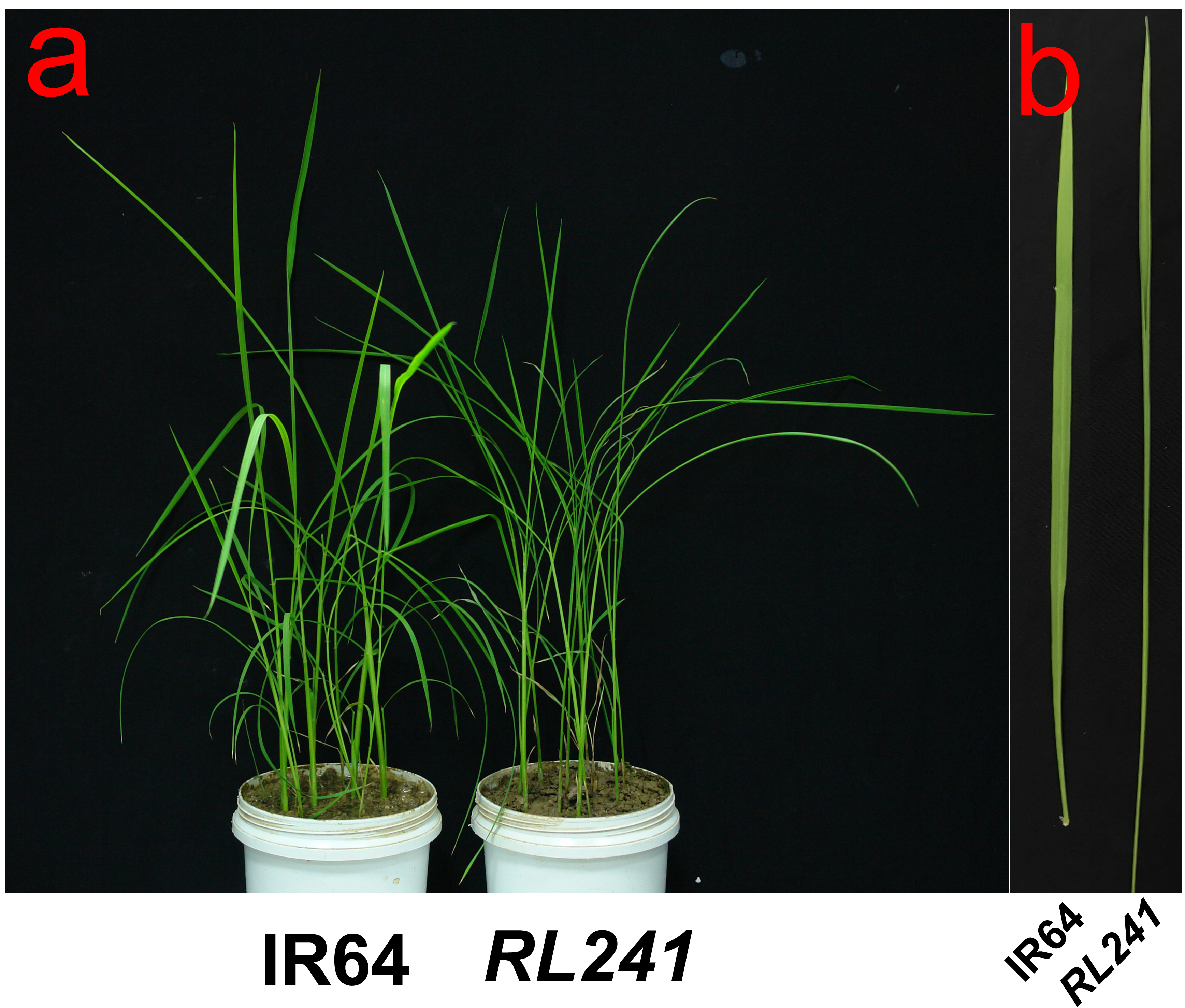

Supplement: Supplementary file 3 — Additional file 3: Figure S3 Leaf mophology of IR64 and its mutant RL241 under control conditions. [file 12284_2020_379_MOESM3_ESM.jpg]

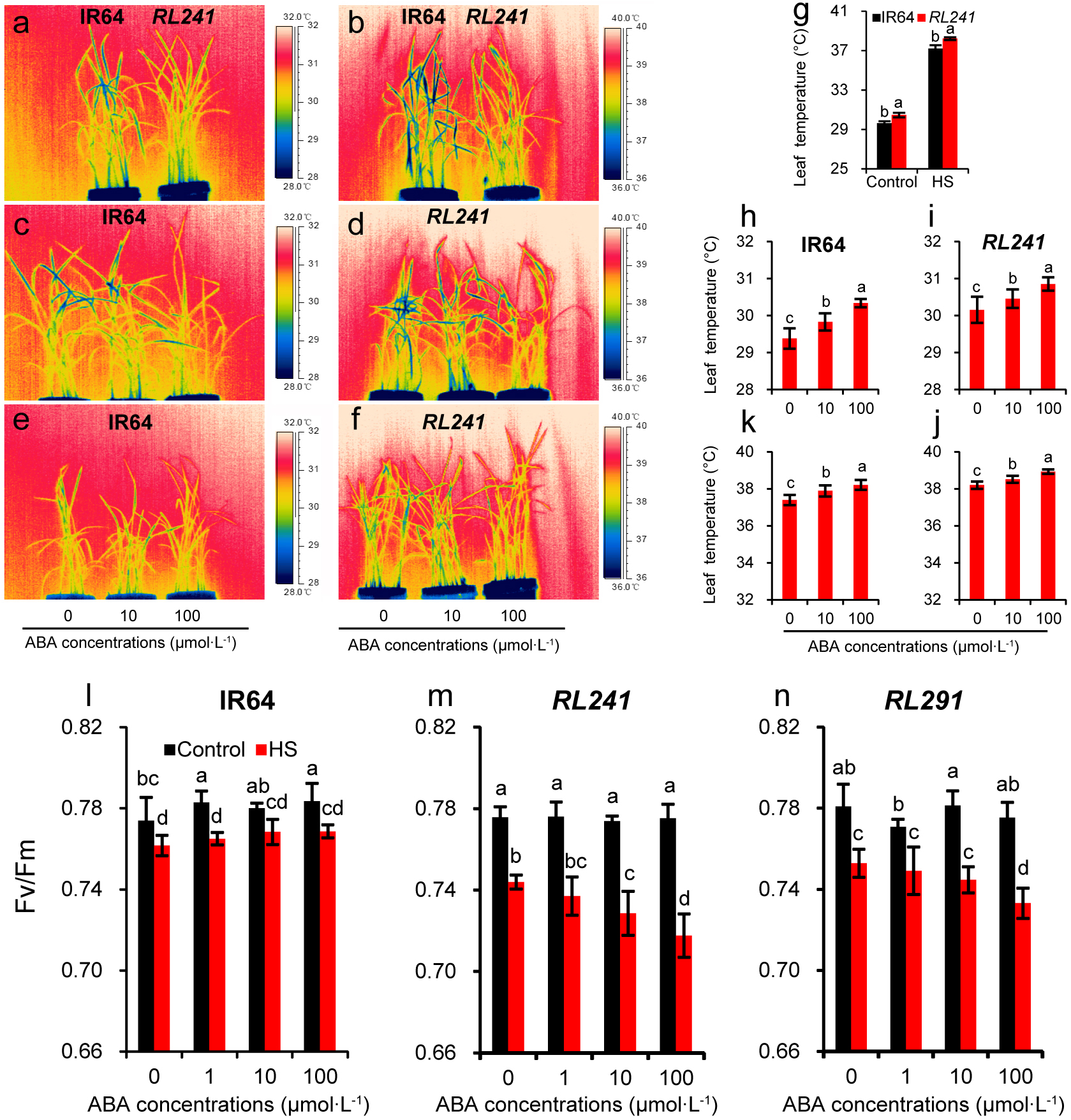

Supplement: Supplementary file 4 — Additional file 4: Figure S4. Effect of ABA on tissue temperature and Fv/Fm of leaves in IR64, RL241 and RL291 plants under heat stress. a and b, Thermal images of rice plants under the heat stress and control treatments without ABA treatment, respectively; c and d, Thermal images of IR64 and RL241 plants under the control with ABA treatments; e and f, Thermal images of IR64 and RL241 plants under heat stress with ABA treatments; g, Leaf temperature of IR64 and RL241 without ABA treatment; h-j; Leaf temperature of IR64 and RL241 with ABA treatments; i-n, Fv/Fm of IR64, RL241 and RL291. Vertical bars denote standard deviations (Tissue temperature, n = 10; Fv/Fm, n = 5). Different letters indicate a significant difference among the ABA treatments under the control and heat-stressed conditions within a genotype by two-way analysis of variance for two factors (temperature and treatment) (P < 0.05). [file 12284_2020_379_MOESM4_ESM.jpg]

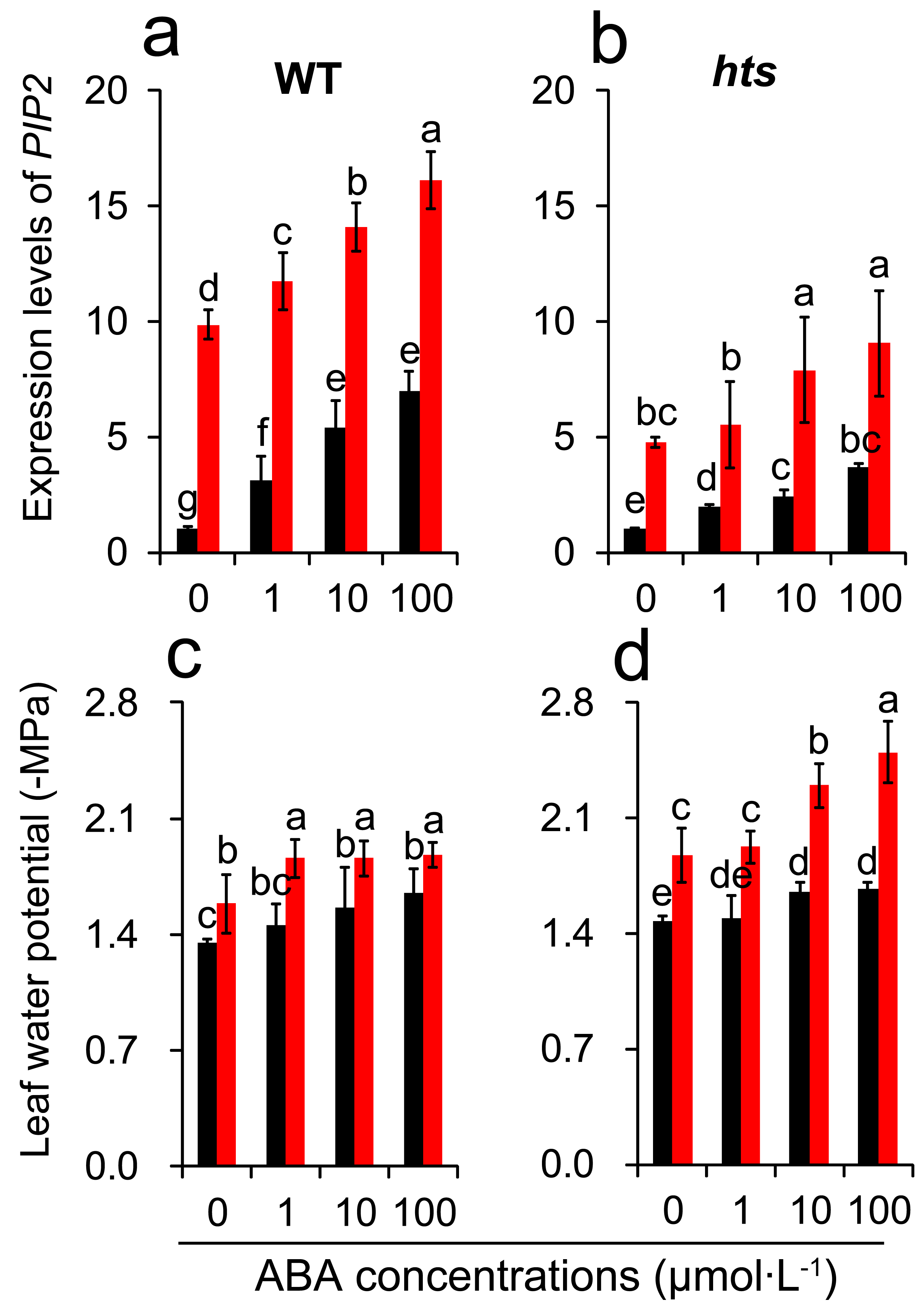

Supplement: Supplementary file 5 — Additional file 5: Figure S5. Effect of ABA on expression levels of the PIP2 gene and water potential of leaves in rice plants under heat stress. a and b, Relative expression of PIP2; c and d, Leaf water potential. Vertical bars denote standard deviations (PIP2, n = 3; Leaf water potential, n = 4). Different letters indicate a significant difference among the ABA treatments under the control and heat-stressed conditions within a genotype by two-way analysis of variance for two factors (temperature and treatment) (P < 0.05). [file 12284_2020_379_MOESM5_ESM.jpg]
