# Supplementary material for: Abscisic Acid Negatively Modulates Heat Tolerance in Rolled Leaf Rice by Increasing Leaf Temperature and Regulating Energy Homeostasis
Source: Rice (N Y). 2020 Mar 13;13:18. doi: 10.1186/s12284-020-00379-3 (PMC7070142; doi:10.1186/s12284-020-00379-3)
Supplement: Supplementary file 6 — Additional file 6: Table S1. Primer sequences used in qRT-PCR. [file 12284_2020_379_MOESM6_ESM.docx]

Table S1 Primer sequences used in qRT-PCR

| Locus ID, gene name | Forward (5’-3’) | Reverse (5’-3’) |
| --- | --- | --- |
| LOC_Os02g58080, *SUT4* | GACTGCCACTCTCGATCACA | ACGATTGCAAGGTTCAGGAC |
| LOC_Os09g08072, *INV1* | AAGAGCAGGGGTGTACAAG | AAGCTGTGAGTCTGTGGCTC |
| LOC_Os02g33110, *CIN1* | CCGGATCTCTACAAGCCAAC | GAAGCTCTCAACCACCGAAC |
| LOC_Os06g09450, *SUS2* | GTGTGCTTGACACCATCCAC | CATGCGGAGACAGGATAACA |
| LOC_Os07g23110, *PARP1* | AGGGCAAGGTTGGTATCACG | GAGCTGGGGGCATCTTTCTT |
| LOC_Os01g24920, *PARP2* | GGACAAGACGAAATCCGCAA | GTCTGGTTCATGGTGGCATC |
| LOC_Os03g16860, *HSP71.1* | CTACGAGGGCATCGACTTCT | CGGTGCTCTTGTCCATCTTG |
| LOC_Os02g52150, *HSP24.1* | TGAGCCTCATGGACGACCT | CCCTTGATCACGAGGCTGTT |
| LOC_Os11g14220, *Tid1* | AGGTTCGATGGTGCTCTGAA | GAAGTGGATCCTCGGGTAGG |
| LOC_Os04g33860, *ACL1* | CCCGTGATCCTTGACTTCCT | CCTGGTCTTCCTGCTGCTAT |
| LOC_Os01g74450, *TIP1* | GGACAACCACTGGGTCTACT | GGCCGATGAAGATGATGTCG |
| LOC_Os01g13130, *TIP4* | GTTGCTGCCGATGACGAAG | GCCGATGAAGACGAAGAGGA |
| LOC_Os04g16450, *PIP2* | ACCGTCATTGGGTACAAGGT | GGTGCAGTAGACGAGGATGA |
| LOC_Os01g22490, *UBQ5* | GACTACAACATCCAGAAGGAGTC | TCATCTAATAACCAGTTCGATTTC |

**Fig.S1** Effect of heat stress on the expression levels of genes associated with bulliform cells in leaves of rice plants. a, Relative expression level of *Tid1*; b, Relative expression levels of *ACL1*. Vertical bars denote standard deviations (n=3). A *t*-test was conducted to compare the difference between control and heat stress within a cultivar. * denotes *P* < 0.05.

**Fig.S2** Effect of heat stress on the expression levels of genes associated with aquaporins in leaves of rice plants. a, Relative expression level of *TIP1*; b, Relative expression level of *TIP4*. Vertical bars denote standard deviations (n=3). A *t*-test was conducted to compare the difference between control and heat stress within a cultivar. * denotes *P* < 0.05.

**Fig.S3** Leaf mophology of IR64 and its mutant RL241 under control conditions.

**Fig.S4** Effect of ABA on tissue temperature and Fv/Fm of leaves in IR64, *RL241* and *RL291* plants under heat stress. a and b, Thermal images of rice plants under the heat stress and control treatments without ABA treatment, respectively; c and d, Thermal images of IR64 and *RL241* plants under the control with ABA treatments; e and f, Thermal images of IR64 and *RL241* plants under heat stress with ABA treatments; g, Leaf temperature of IR64 and *RL241* without ABA treatment; h-j; Leaf temperature of IR64 and *RL241* with ABA treatments; i-n, Fv/Fm of IR64, *RL241* and *RL291*. Vertical bars denote standard deviations (Tissue temperature, n=10; Fv/Fm, n=5). Different letters indicate a significant difference among the ABA treatments under the control and heat-stressed conditions within a genotype by two-way analysis of variance for two factors (temperature and treatment) (*P* < 0.05).

**Fig.S5** Effect of ABA on expression levels of the *PIP2* gene and water potential of leaves in rice plants under heat stress. a and b, Relative expression of *PIP2*; c and d, Leaf water potential. Vertical bars denote standard deviations (*PIP2*, n=3; Leaf water potential, n=4). Different letters indicate a significant difference among the ABA treatments under the control and heat-stressed conditions within a genotype by two-way analysis of variance for two factors (temperature and treatment) (*P*<0.05).
